# Application of bilateral internal mammary artery with different configurations in coronary artery bypass grafting

**DOI:** 10.1186/s13019-020-01380-z

**Published:** 2021-01-06

**Authors:** Zengqiang Han, Guodong Zhang, Shenglong Chen, Gang Liu, Yu Chen

**Affiliations:** Cardiac Surgery Department, Peking University People’s Hospital, BeiJing, 100044 China

**Keywords:** Coronary artery bypass graft, Internal mammary arteries, Transit time flow measurement

## Abstract

**Background:**

A large number of studies have shown that BIMA grafting is superior to single internal mammary artery grafting in cardiac function protection and long-term survival after surgery. While, there is still no consensus on how is the best configuration to use BIMA. This study aims to compare intraoperative blood flow, early clinical results and early postoperative patency of different configurations of BIMA.

**Methods:**

There were 74 patients who underwent CABGs with bilateral internal mammary artery with different configurations we included. According to the different target territories that RIMA grafted to, the patients were divided into bilateral group (group I) with 20 cases and left group (group II) with 54 cases. Intraoperative blood flow, early clinical results and early postoperative patency of different configurations of BIMA were compared.

**Results:**

There was no difference in the early postoperative death and major complications between group I and Group II(*P*>0.05). Compared with the LIMA in group II, the LIMA in group I had a slightly higher DF value (76.7 ± 6.2 vs 73.1 ± 6.8, *P* = 0.040). Compared with the RIMA in group II, the RIMA in group I had a slightly higher MGF (51.7 ± 34.4 ml/min vs 31.4 ± 21.4 ml/min, *P* = 0.024). There was no difference in the other TTFM parameters of LIMA and RIMA between group I and Group II(P>0.05). Further subgroup analysis revealed that compared with free RIMA in group II, in situ RIMA had a higher DF value (71.4 ± 7.8 vs 61.8 ± 18.1,*P* = 0.025). The PI of LIMA in free RIMA subgroup was higher than the PI of LIMA in in-situ RIMA subgroup (3.0 ± 1.6 vs 2.1 ± 1.0,*P* = 0.018). The results of early postoperative CTA examination showed that all IMAs grafts were completely patent.

**Conclusions:**

The use of BIMA for CABG is safe and efficacious, RIMA used in right coronary artery received more satisfactory graft flow. BIMA with no stenosis and occlusion in the early stage, therefore is the ideal and stable coronary bypass graft.

## Background

Since Robert Goetz first performed and published the coronary artery bypass graft surgery in humans in 1961, now CABG has become an important revascularization methods of coronary heart disease [1]. Since the mid-1980s, owing to the high patency, the use of the left internal mammary artery (LIMA) for left anterior descending artery (LAD) grafting has been a cornerstone of CABG surgery [2]. Previous studies showed that the right internal mammal artery (RIMA) has the same anatomical structure and physiological function as the LIMA, and has the same long-term patency rate as LIMA. A large number of studies have shown that BIMA grafting is superior to single internal mammary artery grafting in cardiac function protection and long-term survival after surgery [3–5]. While, owing to high technical requirements, the long operation time, and high incidence rate of sternal wound complications, the use of BIMA grafting is limited, about 5% worldwide, and it is only recommended for younger patients [6]. And there is still no consensus on how is the best configuration to use BIMA [5]. This study aims to compare intraoperative blood flow, early clinical results and early postoperative patency of different configurations of BIMA.

## Material and methods

### Study population

Data for isolated CABG were retrospectively collected from January 1, 2018 to July 31, 2020, from the Peking University People’s Hospital database. Exclusion criteria: (1) coronary artery disease is unsuitable or unnecessary for BIMA;(2) Combined with subclavian artery stenosis;(3) Preoperative IMA ultrasound indicated that the IMA was fine, narrow or calcified;(4) With concomitant additional procedures. There were 74 patients who underwent CABGs with bilateral internal mammary artery with different configurations we included. According to the different target territories that RIMA grafted to, the patients were divided into bilateral group (group I) with 20 cases and left group (group II) with 54 cases. This study was approved by our institutional Review Board /Ethics Committee. Consent for individual use of data was waived because of the nature of the study and previous approval for the use of such data at the time of operative consent.

### Surgical methods

All patients underwent CABGs through a median full sternotomy. Stabilization of the target coronary arteries was accomplished with a tissue stabilizer (Octopus, Medtronic Corporation, Minneapolis, MN) and an intra-coronary shunt (Medtronic Corporation, Minneapolis, MN) was used during off-pump CABGs. In on-pump CABGs, cardiopulmonary bypass was established after standard ascending aorta cannulation and 2-stage venous cannulation of the right atrium, all anastomosis were performed after cardiac arrest. Fifty-six cases of LIMA were harvested by pedicled method, and 18 cases were harvested by the skeletonized method. RIMAs were all harvested by the skeletonized method to maximize the length of the internal mammary artery. After systemic heparinization, the distal end of IMAs were cut, which were protected by poppy alkali gauze.

**Fig. 1:**
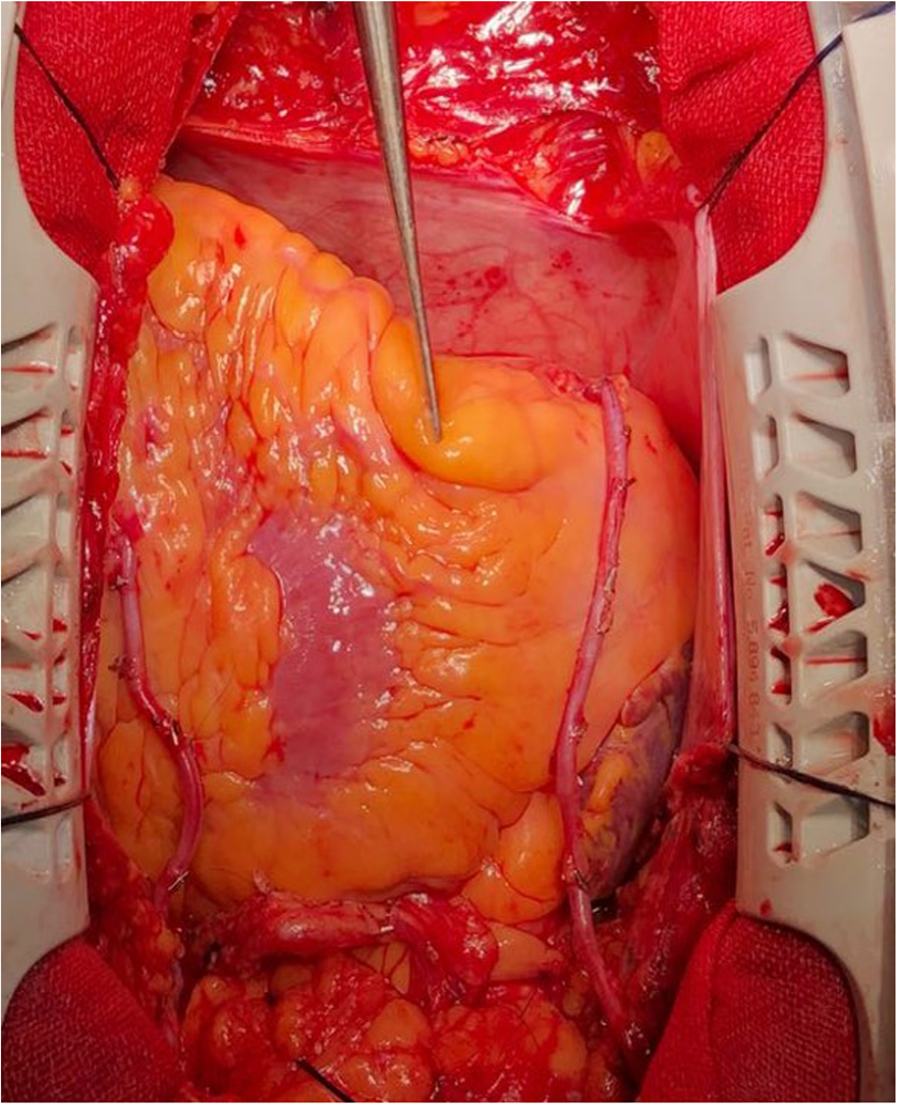
In situ RIMA to right coronary artery or posterior descending artery with in situ LIMA to left anterior descending artery. LIMA, left internal mammary artery; RIMA, right internal mammary artery

BIMA configurations were as follows:

Group I (20 cases): In situ RIMA to right coronary artery or posterior descending artery with in situ LIMA to left anterior descending artery (Fig. 1).

**Fig. 2:**
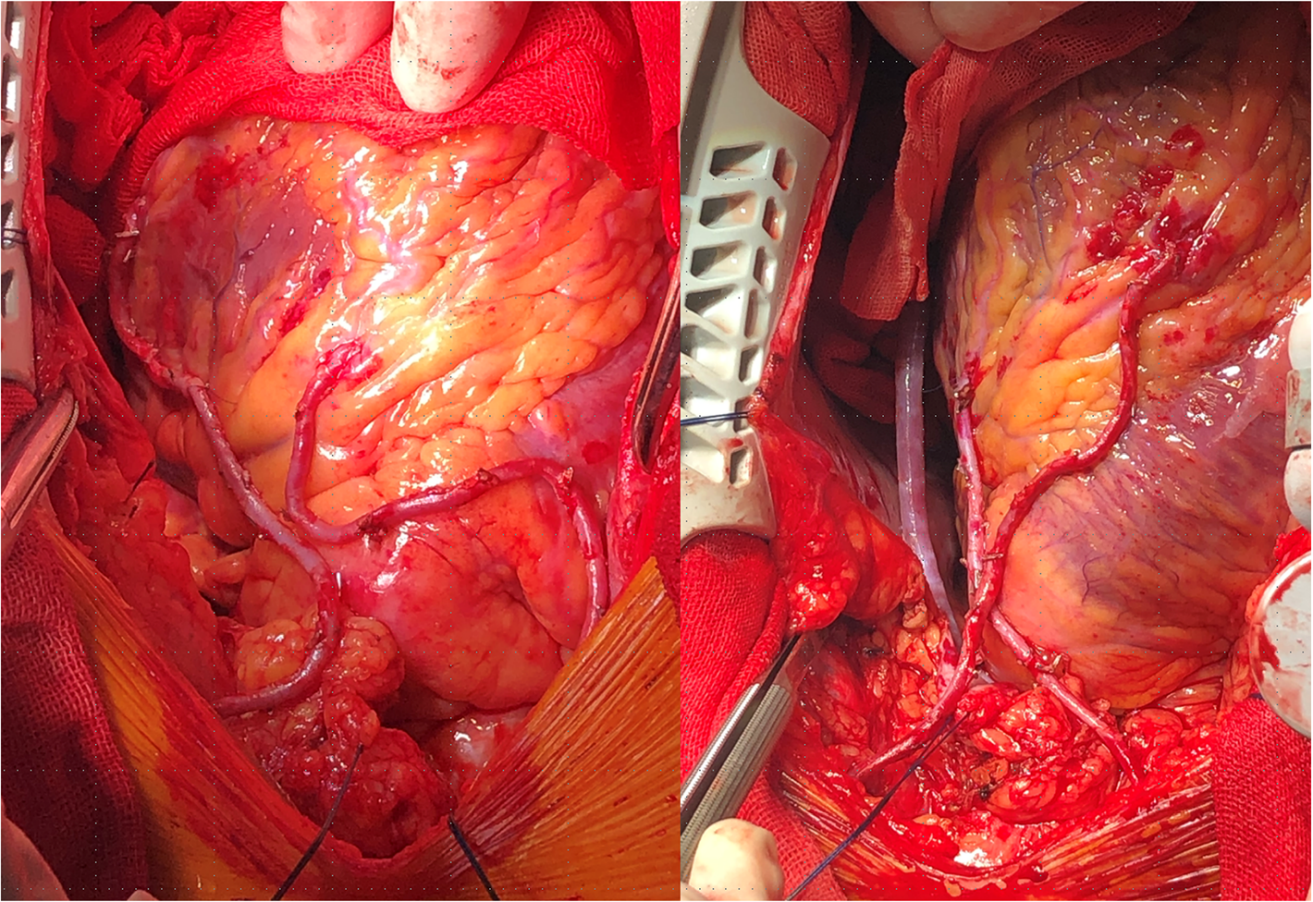
**a** In situ RIMA to LAD with in situ LIMA to diagonal branch. **b** In situ RIMA to LCX with in situ LIMA to LAD. LAD, left anterior descending artery; LIMA, left internal mammary artery; RIMA, right internal mammary artery; TTFM, transit-time flow measurement

Group II (54 cases) was divided into: (1) In situ RIMA to LAD with in situ LIMA to diagonal branch (D) or left circumflex branch (LCX)(17 cases) (Fig. 2a).(2) In situ RIMA to D or LCX with in situ LIMA to LAD (14 cases) (Fig. 2b).(3) In situ LIMA to LAD and LIMA-Y-free RIMA to D or LCX (13 cases).(4) In situ LIMA to LAD with aorta-free RIMA to D or LCX (10 cases). Other target territories were revascularized by the great saphenous vein or radial artery.

### Preoperative internal mammary artery ultrasonography examination

Preoperative blood flow parameters of IMAs were measured by transthoracic doppler ultrasonography machine (APLIO500 TUS-A500, Probe: PLT-704SBT and PVT-712BT). All preoperative internal mammal artery ultrasonography was performed by the same senior ultrasonologist.

### Intraoperative transit time flow measurement

The transit time flow of the grafts was measured by The VeriQ system TTFM device (MediStim Inc., Oslo, Norway), equipped with2,3 or 4-mm probes, depending on the size of the graft, under stable haemodynamic conditions without the support of a mechanical device such as cardiopulmonary bypass or an intra-aortic balloon pump. The parameter yielded by TTFM system including(i) the mean graft flow volume (MGF, ml/min), (ii) the pulse index calculated as (PI, maximum flow volume—minimum flow volume)/(mean flow volume) and (iii) the DF, calculated as (flow volume of the diastolic phase)/(flow volume of the systolic phase + flow volume of the diastolic phase). Satisfactory blood flow parameters criteria: 1,ACI>50%;2,The shape of blood flow waveform is stable and repeatable; 3,PI<5, MGF>15 ml/min. If sufficient graft flow was not obtained, graft revision was considered and performed until diastolic graft flow was confirmed.

### Postoperative management

Postoperatively, aspirin, nitroglycerine and β- blocker were prescribed on postoperative day 1. The patients were routinely examined by cardiac CT scanning prior to discharge from the hospital unless they had grade 3 or more chronic kidney disease.

### Statistical analysis

The database was established by EpiDate3.1 software, the data were input twice in parallel. The final analysis database is formed after logical error checking and sorting of the input data and analysis and processing of outliers. Continuous variables were expressed as means ±SDs; if the data conformed to a normal distribution, the two groups were compared using an independent samples t test, and multiple groups were compared using variance analysis. For nonnormally distributed data, Wilcoxon rank-sum tests were used for comparisons between two groups, and Kruskal-Wallis H tests were used for comparison between multiple groups. Categorical variables were described as percentages (rates); comparisons between two groups were performed using Pearson’s χ2 test or Fischer’s exact test as appropriate. *P* < 0.05 was considered statistically significant. All analyses were performed in SPSS version 23.

## Results

Baseline patient characteristics shown in Table 1. There was no difference in the baseline patient characteristics between group I and Group II(P>0.05).

**Table 1:** Demographic and clinical characteristics

Items	Group I(*n* = 20)	Group II(*n* = 54)	P
Gender			1.000
Male	18 (90.0%)	49 (90.7%)	
Female	2 (10.0%)	5 (9.3%)	
Age	58.2 ± 8.9	56.4 ± 10.4	0.492
BMI (kg/m2)	25.6 ± 2.8	25.6 ± 3.1	0.952
Diabetes (n, %)	9 (45.0%)	16 (29.6%)	0.214
Hypertension (n, %)	11 (55.0%)	30 (55.6%)	0.966
Hyperlipidemia (n, %)	8 (40.0%)	18 (33.3%)	0.594
PVD (n, %)	1 (5.0%)	7 (13.0%)	0.435
previous PCI	5 (25.0%)	4 (7.4%)	0.054
COPD	1 (5.0%)	1 (1.9%)	0.470
LM disease	2 (10.0%)	8 (14.8%)	0.719
LVEF(%)	61.9 ± 10.7	62.0 ± 10.6	0.971
Cr (mol/L)	72.8 ± 12.9	76.6 ± 16.0	0.236

*BMI* body mass index, *COPD* chronic obstructive pulmonary disease, *PVD* peripheral vascular diseases, *LVEF* left ventricular ejection fraction, *LM* left main disease

### The comparison of early postoperative death and major complications between group I and group II

One case died of low cardiac output syndrome due to perioperative myocardial infarction early postoperatively in group II (1.35%). There was no difference in total operation time (5.2 ± 1.2 vs 5.5 ± 1.2 h,*P* = 0.457) between group I and Group II, however the heparin-protamine time of group II was longer than that of group I (138.9 ± 41.0 vs173.4 ± 58.9 min,*P* = 0.019). There was no difference in the early postoperative death and major complications between group I and Group II(P>0.05) (Table 2).

**Table 2:** Baseline procedural characteristics of group I and group II

Items	Group I(*n* = 20)	Group II(*n* = 54)	P
Operation time(h)	5.2 ± 1.2	5.5 ± 1.2	0.457
Heparin protamine time (min)	138.9 ± 41.0	173.4 ± 58.9	0.019
Off-pump	13 (65.0%)	36 (66.7%)	0.893
Number of anastomosis	3.2 ± 0.9	3.3 ± 0.8	0.621
Perioperative MI	1 (5.0)	2 (3.7%)	1.000
CRRT (n, %)	1 (5.0%)	7 (13.0%)	0.435
Volume of blood (ml)	760.0 ± 373.3	772.2 ± 516.1	0.923
RBC transfusion(U)	2.1 ± 2.4	2.4 ± 4.2	0.738
Re- thoracotomy	0 (0)	2 (3.7%)	1.000
Sternal complication	1 (5.0%)	1 (1.9%)	0.470
Ventilator time(h)	17.1 ± 17.2	22.8 ± 42.4	0.567
ICU stay time(h)	43.9 ± 39.5	46.7 ± 54.6	0.835
Post-operative stay time(d)	10.7 ± 5.0	11.1 ± 7.5	0.827

*CRRT* continuous renal replacement therapy, *RBC* red blood cell, ICU intensive care unit

### The comparison of intraoperative blood flow parameters between group I and group II

Compared with the LIMA in group II, the LIMA in group I had a slightly higher DF value (76.7 ± 6.2 vs 73.1 ± 6.8, *P* = 0.040). Compared with the RIMA in group II, the RIMA in group I had a slightly higher MGF (51.7 ± 34.4 ml/min vs 31.4 ± 21.4 ml/min, *P* = 0.024). There was no difference in the other TTFM parameters of LIMA and RIMA between group I and Group II(P>0.05) (Table 3). Further subgroup analysis revealed that compared with free RIMA in group II, in situ RIMA had a higher DF value (71.4 ± 7.8 vs 61.8 ± 18.1,*P* = 0.025). The PI of LIMA in free RIMA subgroup was higher than the PI of LIMA in in-situ RIMA subgroup (3.0 ± 1.6 vs 2.1 ± 1.0,*P* = 0.018) (Table 4).

**Table 3:** The comparison of TTFM parameters between Group I and Group II

TTFM	Group I(*n* = 20)	Group II(*n* = 54)	P
LIMA-MF	30.7 ± 10.3	32.3.8 ± 22.3	0.671
LIMA-PI	2.2 ± 0.6	2.5 ± 1.4	0.288
LIMA-DF	76.7 ± 6.2	73.1 ± 6.8	0.040
RIMA-MF	51.7 ± 34.4	31.4 ± 21.4	0.024
RIMA-PI	2.1 ± 1.1	2.7 ± 2.5	0.307
RIMA-DF	64.8 ± 6.8	67.2 ± 14.0	0.332
BIMA-MF	78.0 ± 42.7	62.2.1 ± 37.8	0.128

*TTFM* transit time flow measurement, *LIMA* left internal mammary artery, *RIMA* left internal mammary artery, *MGF* mean graft flow, *PI* pulse index, *DF* diastolic flow fraction

**Table 4:** The comparison of TTFM parameters between in situ RIMA and free RIMA

TTFM	In situ RIMA(*n* = 31)	Free RIMA(*n* = 23)	P
LIMA-MF	35.9 ± 24.7	27.4 ± 18.0	0.168
LIMA-PI	2.1 ± 1.0	3.0 ± 1.6	0.018
LIMA-DF	72.1 ± 6.2	74.5 ± 7.5	0.203
RIMA-MF	33.4 ± 24.1	28.9 ± 17.2	0.453
RIMA-PI	2.3 ± 0.6	3.2 ± 3.7	0.223
RIMA-DF	71.4 ± 7.8	61.8 ± 18.1	0.025
RIMA-MF	66.7 ± 45.1	56.3 ± 24.4	0.282

*TTFM* transit time flow measurement, *LIMA* left internal mammary artery, *RIMA* left internal mammary artery, *MGF* mean graft flow, *PI* pulse index, *DF* diastolic flow fraction

### Results of the CT angiography examined before discharge

A total of 74 patients were examined for coronary CT angiography before discharge. The results of early postoperative CTA examination showed that all IMAs grafts were completely patent.

## Discussion

Arterial conduits, including the left internal mammal artery [7], bilateral internal mammal arteries [8], and the radial artery have excellent patency [9], resulting in better long-term survival in comparison with CABG using the saphenous vein graft. The strategy of in situ LIMA grafting to the LAD (LIMA-LAD) is considered the “gold standard” of coronary revascularization [10]. Previous studies proved a substantial survival benefit from both right internal mammary artery and radial artery used as the second conduit compared with single internal mammary artery plus saphenous vein graft [11, 12]. Owing to not need extra incisions and the advantages of not being spasmodic, RIMA has been the first choice as the second arterial conduit. However, due to high technical requirements, the long operation time, and high incidence rate of sternal wound complications, the use of BIMA grafting is limited, about 5% worldwide, and it is only recommended for younger patients [6]. From 1999 to 2009, the application proportion of BIMA in the United States was stable at around 4%, with almost no significant change [13]. The application proportion of BIMA in Europe was also less than 10% [14]. We used BIMA to perform CABG in 74 patients and achieved satisfactory short-term efficacy, but the long-term effect still needs further follow-up observation. BIMA harvested by the skeletonized method can better retain collateral vessels that supply the sternum, which might help reduce the probability of nonunion and deep infection of the sternum [15]. In our study, 56 cases of LIMA were harvested by pedicled method, and 18 cases were harvested by the skeletonized method. RIMAs were all harvested by the skeletonized method. Minor sternal incision complications occurred in 2 patients, including 1 diabetic patient with BMI of 27.7 and the other case without diabetes but had a BMI of 34.3. There was no deep sternal infection in 74 cases. We suggest that RIMA should be harvested by skeletonized method because of its long walking path. At the same time, LIMA can be routinely obtained with pedicle method in most patients, which reduce the operation time and does not increase the risk of sternal complications. BIMA should be routinely harvested by the skeletonized method in patients with obesity, diabetes, or severe respiratory disease. During the operation, attention should be paid to avoid sternal fracture caused by excessive retraction of sternal retractor and to strengthen the protection of sternal incision. Sternal ligation belt should be used as far as possible to reduce postoperative sternal cutting, and “8” suture should be used when using steel wire. More attention should be paid to the strict monitoring and control of blood glucose after operation.

At present, there is still no consensus about the optimal configuration of BIMA, different groups configurations may affect the short-and long-term outcomes of patients. The lack of evidence for the strategy of configuration makes the choice of BIMA more uncertain, which limits its promotion. Sheila et al. reported that the long-term outcome of BIMA used for revascularization in the left coronary territories was better than that in left and right coronary territories respectively. The possible explanation may be that the left coronary is more important than the right coronary system [16]. The graft failure occurred in left coronary system has more significant negative effect than that occurred in right coronary system. However, this study showed that the blood flow was better when BIMA was used in the left and right coronary arteries respectively than when BIMA was used in the left coronary arteries only, which may related to that about 80% Chinese people are right-coronal dominant type. Previous studies have showed that in situ RIMA used for the left coronary system increases the difficulty second operation. This study confirmed that RIMA can also achieve satisfactory blood flow in the right coronal system. A meta-analysis reported by Yanagawa et al., which included 8 studies, showed that the short-term and long-term outcomes of in situ BIMA and composite “Y” conduit were similar, but the results were significantly different between different studies [17]. In this study, in situ LIMA was used in all cases, and group II was divided into two subgroups, in situ and free RIMA, comparison between subgroups showed that in situ RIMA could achieve better blood flow, which was consistent with previous literature reports [18]. When BIMA was completely used in the left coronary system, there were no statistically significant differences between LIMA-LAD and RIMA-LAD in terms of MF, PI or DF. When the length of RIMA was not enough, free RIMA was used to form a “Y -shaped” configuration with in situ LIMA [13] or directly anastomosed to the ascending aorta [2] to revascularize the D or LCX. There was no significant difference between the two groups in terms of PI, MGF and DF. When the proximal diameter of the RIMA is thick enough, it can be directly anastomosed to the ascending aorta to avoid the uncertain impact on the LIMA-LAD. A total of 74 patients were examined for coronary CT angiography before discharge and showed that all IMAs grafts were completely patent.

## Limitations

Several limitations of our study should be recognized. The first and most important limitation of this study was its descriptive nature, using a relatively small cohort of patients at a single institution. Second, the lack of long-term follow-up data was a limitation.

## Conclusions

The use of BIMA for CABG is safe and efficacious, RIMA used in right coronary artery received more satisfactory graft flow. BIMA with different configurations and harvest method can be choose according the different target conditions.

## Data Availability

Data will be made available on request.

## Abbreviations

LIMA: Left internal mammary artery
LAD: Left anterior descending artery
CABG: Coronary artery bypass graft surgery
TTFM: Transit-time flow measurement
PI: Pulse index
MGF: Mean graft flow
DF: Diastolic flow fraction
SVG: Great saphenous vein
CTA: Computed tomography angiography
